# Elevated Global SUMOylation in Ubc9 Transgenic Mice Protects Their Brains against Focal Cerebral Ischemic Damage

**DOI:** 10.1371/journal.pone.0025852

**Published:** 2011-10-07

**Authors:** Yang-ja Lee, Yongshan Mou, Dragan Maric, Dace Klimanis, Sungyoung Auh, John M. Hallenbeck

**Affiliations:** 1 Stroke Branch, National Institute of Neurological Disorders and Stroke (NINDS), National Institutes of Health (NIH), Bethesda, Maryland, United States of America; 2 Laboratory of Neurophysiology, National Institute of Neurological Disorders and Stroke (NINDS), National Institutes of Health (NIH), Bethesda, Maryland, United States of America; 3 Clinical Neurosciences Program (HNQ22), National Institute of Neurological Disorders and Stroke (NINDS), National Institutes of Health (NIH), Bethesda, Maryland, United States of America; Charité Universitaetsmedizin Berlin, Germany

## Abstract

We have previously shown that a massive increase in global SUMOylation occurs during torpor in ground squirrels, and that overexpression of Ubc9 and/or SUMO-1 in cell lines and cortical neurons protects against oxygen and glucose deprivation. To examine whether increased global SUMOylation protects against ischemic brain damage, we have generated transgenic mice in which Ubc9 is expressed strongly in all tissues under the chicken β-actin promoter. Ubc9 expression levels in 10 founder lines ranged from 2 to 30 times the endogenous level, and lines that expressed Ubc9 at modestly increased levels showed robust resistance to brain ischemia compared to wild type mice. The infarction size was inversely correlated with the Ubc9 expression levels for up to five times the endogenous level. Although further increases showed no additional benefit, the Ubc9 expression level was highly correlated with global SUMO-1 conjugation levels (and SUMO-2,3 levels to a lesser extent) up to a five-fold Ubc9 increase. Most importantly, there were striking reciprocal relationships between SUMO-1 (and SUMO-2,3) conjugation levels and cerebral infarction volumes among all tested animals, suggesting that the limit in cytoprotection by global SUMOylation remains undefined. These results support efforts to further augment global protein SUMOylation in brain ischemia.

## Introduction

With accelerating discovery of new brain injury mechanisms (reviewed in: [1], [2]) and continuing failure of clinical trials of cell-based therapies targeting single mechanisms [3], ischemic brain damage has gradually become more widely viewed as a highly complex, multifactorial process that involves the interplay of many non-dominant effectors [4], [5]. In order to confront the enormous biocomplexity of network dynamics in acute stroke and until Systems Biology provides solutions, our lab focused on broadly plurifunctional targets that maintain homeostasis in states of tolerance to brain ischemia. A candidate that meets these criteria is global SUMOylation, a potentially “drugable” form of post-translational modification with the Small Ubiquitin-like MOdifer that appears to have widespread beneficial effects in the dynamic network, operates in states of tolerance, and acts to preserve homeostasis under stress [6].

SUMO, like ubiquitin, is synthesized as an inactive precursor, and processed by SUMO-specific proteases to yield the mature di-glycine C-terminus. A single heterodimeric E1 activating enzyme (SAE1/SAE2) initiates conjugation by adenylating SUMO, followed by the formation of a covalent thioester E1-SUMO intermediate. SUMO is then transferred to the catalytic cysteine of the single E2 conjugase, Ubc9 (Ubiquitin conjugase 9), which alone or in concert with an E3 ligase catalyzes the formation of an isopeptide linkage between the C-terminal glycine residue of SUMO and the epsilon-amino group of the substrate lysine residue. Isopeptidases catalyze de-SUMOylation and thus balance Ubc9 conjugation activities to modulate steady state levels of SUMO-conjugates (reviewed in [7], [8]). There are three systemically distributed SUMO paralogs in mammals. Two, SUMO-2 and SUMO-3, are 96% identical in amino acid sequence and are difficult to distinguish. In contrast, SUMO-1 is only 45% identical with the other two SUMO paralogs and has distinct immunoreactivity [9].

We initially examined changes in SUMOylation levels during the hibernation cycle in 13-lined ground squirrels (*Spermophilus tridecemilineatus*) that rank among the most brain hypoperfusion-resistant mammals known [10]. During torpor, these animals reduce their brain blood flow levels to roughly 10% of baseline values for up to weeks at a time. Upon arousal, they show no cellular damage in the brain [10], [11], [12] despite prolonged brain perfusion levels characteristic of the “ischemic core” [13], a tissue zone that is generally regarded as unsalvageable in a stroke. We found a massive 10- to 30-fold increase in global SUMOylation (“global” here denotes increased band density within a defined molecular weight range on immunoblots, not that all proteins in those bands have increased SUMOylation) in many squirrel tissues during torpor involving both SUMO-1 and SUMO-2/3 paralogs [14]. Despite suppression of protein translation in hibernating squirrels to roughly 1/10,000^th^ of baseline rates [15], levels of the SUMO conjugase, Ubc9, rose to around three-fold above baseline during torpor [14]. Additional *in vitro* ischemia studies conducted in cell lines and primary cortical neuron cultures exposed to OGD confirmed that increased global SUMOylation supports cell survival and OGD tolerance [16]. Ischemic challenge increases SUMOylation, particularly with SUMO-2,3, in various models: rat focal ischemia [17], [18], mouse focal ischemia [18], rat global ischemia [19], and *in vitro* ischemic models [20], [21]. It is not yet clear whether the increase of SUMO-2,3-ylation during ischemic insults is a result of damage or an active host defense system. At least, recent data published by Datwyler et al. [22] appears to support an endogenous neuroprotective impact of SUMO-2,3 conjugation in an *in vitro* ischemic model.

Based on the foregoing facts and findings, we have now generated Ubc9 transgenic mice, in which Ubc9 expression is increased in all tissues at levels that range among the various lines from 2 to 30 times the endogenous level. Up to five-fold increases in Ubc9 produced corresponding increases in global SUMO-1 and SUMO-2,3 conjugation levels. A highly significant inverse relationship among all transgenic lines was noted in which the greater were the levels of global SUMOylation, the less was the extent of infarct damage in the brains of animals exposed to permanent middle cerebral artery occlusion (pMCAO) for 24 hours.

## Results

### Generation of Ubc9 transgenic mice

**Figure 1:**
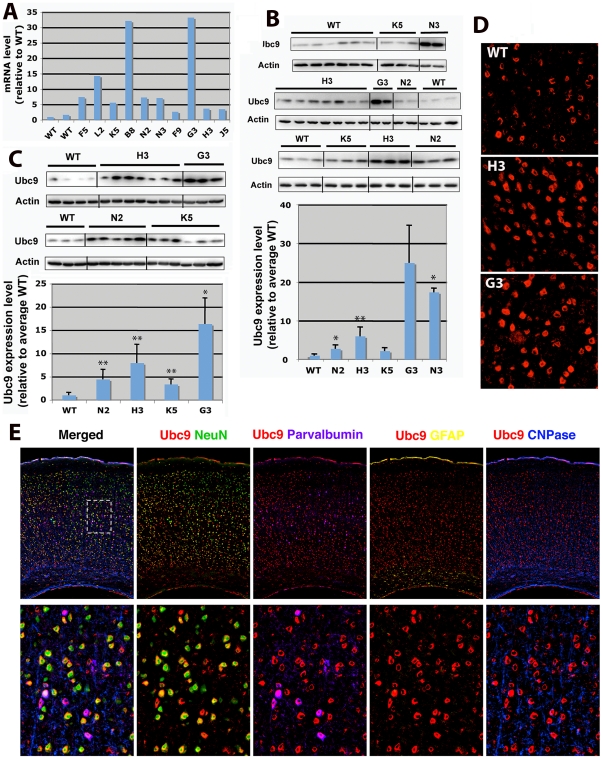
Ubc9 expression levels in WT and Ubc9 transgenic mice. (A) Ubc9 message levels in tail sample from WT and Ubc9 transgenic founder lines (first generation) were measured in triplicate by real-time PCR. The average Ubc9 mRNA level in each animal was normalized by its tubulin mRNA level and plotted relative to the WT average level. (B) Ubc9 protein levels in tail samples. Protein (20 µg) isolated from tail samples from WT and Ubc9 transgenic mice was analyzed by Western blot (upper panel). Intensities of Ubc9 protein bands were normalized to actin and plotted relative to WT average level (lower panel). n = 2–10 (mean±SD). *P<0.05, **P<0.01 (note: G3 n = 2 with large SD). (C) Ubc9 protein levels in brain samples. Proteins (20 µg each) isolated from brains of WT and transgenic mice were analyzed by Western blot (upper panel). Intensities of Ubc9 protein bands were normalized to actin and plotted relative to WT average level (lower panel). n = 3–7 (mean±SD). *P<0.05, **P<0.01. (D) Ubc9 expression levels in brain tissue (cerebral cortex, Layer III-external pyramidal region) of WT and Ubc9 transgenic mice (H3, G3). Ubc9 was shown in red. (E) Ubc9 expressing cells in the cerebral cortex of Ubc9 transgenic mouse (line H3). Upper panels show all layers of cerebral cortex, and lower panels show an enlarged region of the Layer III-External pyramidal area (dashed rectangle in the upper merged panel). NeuN (green), a pan-neuronal marker; parvalbumin (purple), a marker for interneurons; GFAP (yellow), a marker for astrocytes; CNPase (blue), a marker for oligodendrocytes. Ubc9 is shown in red.

The Ubc9 gene was transferred into mice by pronucleus injection. Of 98 potential founder animals, Southern blot analysis revealed 10 animals (B8, F5, F9, G3, H3, K5, J5, L2, N2, N3) carrying the transgene. The Ubc9 expression (mRNA) levels in tail samples from these founder lines were analyzed by real-time PCR. The expression levels varied greatly among these lines as shown in Figure 1A. Transgenic lines B8 and G3 are the highest Ubc9 expressing lines, followed by L2, F5, N2 and N3, and the rest of the lines showed only a few-fold increase compare to wild type animals. Some of the lines (B8, F5, F9, J5 and L2) did not breed well, and were eventually lost. Inability to breed was not necessarily correlated with Ubc9 expression level, since one of the highest Ubc9 expressing line, G3, was still able to breed. Among surviving lines, we examined Ubc9 protein levels in tail samples by Western blot. As shown in Figure 1B, the line G3 showed the highest protein expression level followed by lines N3 and H3. The lines N2 and K5 showed only a few-fold increase. The Ubc9 expression levels in brains (Fig. 1C) among transgenic lines were very similar to those in tail samples. Similar Ubc9 expression levels were observed in kidneys as well (data not shown), suggesting that the gene was expressed systemically. Elevated Ubc9 expression in transgenic mice was also confirmed by immunostaining of the brain tissue (Fig. 1D). Ubc9 expression was not only higher, but also more condensed in cell nuclei in transgenic (H3, G3) mouse brain compared to wild type mouse brain. Ubc9 was expressed predominantly in neurons (NeuN: Neuronal Nuclei positive and parvalbumin positive) in the brain (Fig. 1E shows cerebral cortex and Fig. S1 shows a whole brain coronal section).

### Pathology, physiology and phenotype of Ubc9 overexpression

Six 21-week old animals (3 male and 3 female) each from WT, low Ubc9 expressing lines (N2, K5 and some of H3) and high Ubc9 expressing lines (G3 and some of H3) were subjected to pathology and phenotyping analyses. There are no differences in hematological analysis or serum chemistry among these animals. No gross abnormal findings including tumors were noted in any of the WT mice or in any of the Ubc9 transgenic mice. There were no significant differences in average blood gas levels between WT and Ubc9 transgenic (Tg) mice: pH 7.41±0.02 (WT), pH 7.40±0.02 (Tg); PCO_2_ level (mmHg): 42.6±4.2 (WT), 43.2±1.9 (Tg); PO_2_ level (mmHg): 127.0±17.6 (WT), 127.1±16.0 (Tg). Body temperatures of all animals were between 36.5–37.5 °C. Blood pressure was also monitored during the surgical procedure, and there was no significant difference between the average mean arterial blood pressures in WT and Tg animals: (mmHg): 83±6 (WT), 80±8 (Tg). There was also no difference in baseline ipsilateral brain blood flows measured by a laser doppler perfusion monitor (PERIMED) and expressed as PeriFlux Units (PU): 103.4±21.4 (WT), 97.0±35.5 (Tg). Similarly, the percent reduction of ipsilateral brain blood flows during the pMCAO surgical procedure did not differ: 75±6% (WT), 77±5% (Tg).

### Ubc9 transgenic mice are more tolerant to ischemia than WT mice

**Figure 2:**
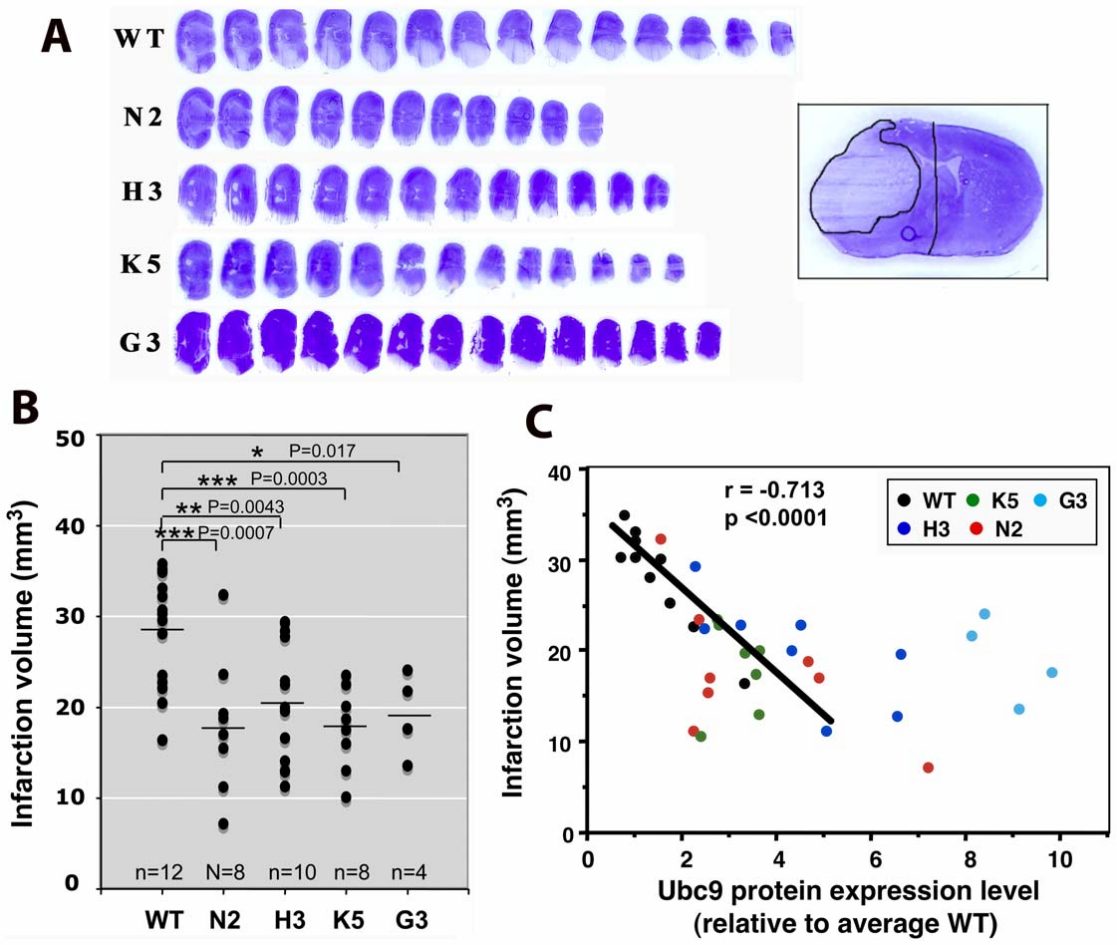
Ubc9 transgenic mice are more tolerant to ischemia than WT mice. (A) Examples of cresyl violet stained coronal brain sections from WT and Ubc9 transgenic mice (N2, H3, K5 and G3) after 24 h pMCAO. The inset shows an enlarged section with a clear infarcted area for a precise measurement. (B) Scatter plot of brain infarction volumes in WT (n = 12) and Ubc9 transgenic mice, N2 (n = 8), H3 (n = 10), K5 (n = 8) and G3 (n = 4) after 24 h pMCAO. Average infarction volume in each animal group is shown as a short horizontal line. *P<0.05, **P<0.01, ***P<0.001. (C) The brain infarction volumes (y-axis) after 24 h pMCAO are plotted against their Ubc9 protein levels (x-axis). A correlation is seen among animals whose Ubc9 levels are under 5-fold of wild type. (Pearson correlation coefficient) r = −0.713, p<0.0001.

Since Ubc9 high-expressing lines, G3 and N3, produced pups less frequently, we mainly used transgenic lines whose Ubc9 levels were elevated only several fold (N2, H3, K5) compared to wild type (WT) animals, but also included a few of the very high expressing line, G3, for pMCAO surgery. The infarction volumes in brains from WT and Ubc9 transgenic mice were quantified at 24 h after pMCAO. Typical cresyl violet staining of the 20 micron coronal brain sections of WT and transgenic mice after 24 h pMCAO are shown in Figure 2A. Brain infarction volumes were calculated by summing cross-sectional areas and multiplying these areas by the distance between the affected sections followed by correction for brain swelling (edema). The average brain infarction volumes (mm^3^) caused by 24 h pMCAO were 28.4±6.0 (WT), 17.8±6.8 (N2), 20.8±6.5 (H3), 16.8±4.6 (K5) and 19.3±4.6 (G3) (mean±SD) (Fig. 2B). All four transgenic lines (N2, H3, K5, G3) used in this experiment had significantly smaller average brain infarctions compared to WT mice. When we plotted infarction volume (y-axis) against Ubc9 expression level in the brain (x-axis) for animals that had been subjected to pMCAO, infarction volumes showed a highly significant inverse correlation with the Ubc9 expression levels (r, Pearson correlation coefficient = −0.731, p<0.0001), when transgenic animals with Ubc9 expression levels up to a threshold of 5-fold higher than those of wild type animals were compared (Fig. 2C). Above this threshold, the correlation was no longer linear.

### The SUMO-1 and SUMO-2,3 conjugation levels are increased in Ubc9 transgenic mice with and without MCAO

**Figure 3:**
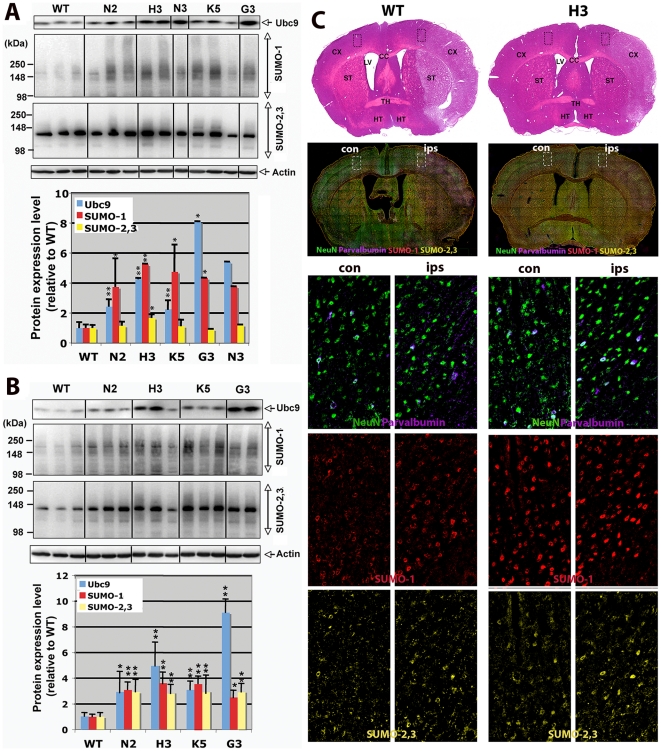
SUMO-1 and SUMO-2,3 conjugation levels in brains of WT and Ubc9 transgenic mice with and without MCAO surgery. (A) Top panel: A representative immunoblot of high molecular weight (HMW) (100∼300 kDa) conjugates of SUMO-1 and SUMO-2,3 along with Ubc9 and actin in brain samples from WT and Ubc9 transgenic (N2, H3, N3, K5, G3) mice without MCAO surgery. Bottom panel: Quantitative analysis of Ubc9, SUMO-1 HMW-conjugates and SUMO-2,3 HMW-conjugates. Intensities were measured, normalized to actin in each sample, and plotted relative to WT average level. n = 3–5 per group (mean±SD; except N3, n = 1). *P<0.05, **P<0.01. (B) Comparable immunoblot (top panel) and quantitative analysis (bottom panel) of Ubc9, SUMO-1 and SUMO-2,3 HMW-conjugates from mice after MCAO surgery. Data are shown as the mean with standard deviation of 4–10 samples in each animal group. *P<0.05, **P<0.01. (C) Immunofluorescence analysis of SUMO-1 and SUMO-2,3 protein distributions in infarcted brains. Top row, H&E staining of coronal brain sections from WT and Ubc9 transgenic (H3) mice after 24 h pMCAO. CX, cerebral cortex; CC, corpus callosum; LV, lateral ventricle; ST, striatum; TH, thalamus; HT, hypothalamus. The second row from the top, comparable coronal brain sections from WT and H3 mice immunostained for SUMO-1 (red), SUMO-2,3 (yellow), NeuN (green) and parvalbumin (purple). con, contralateral side; ips, ipsilateral side. Bottom three sets of paired panels show comparable regions of the layer III-external pyramidal area (enlarged from dashed areas in the upper panels).

We checked whether the Ubc9 upregulation by the transgene increases SUMO conjugation levels. The SUMO conjugation levels, especially the high molecular weight (HMW) (MW>100 kDa) conjugates, were generally higher in transgenic mouse lines (N2, H3, K5, G3) compared with WT (representative immunoblots are shown in Fig. 3A, top panel) (whole immunoblots are shown in Fig. S2). Densities of Ubc9 protein bands, SUMO-1 HMW conjugates and SUMO-2,3 HMW conjugates in several immunoblots (n = 5 for WT, n = 4 for N2, n = 5 for H3, n = 5 for K5, n = 3 for G3 and n = 1 for N3) were measured, normalized by the corresponding actin levels, and expressed as density relative to the WT average (Fig. 3A, bottom panel). SUMO-1 HMW conjugates levels are significantly higher (3∼6-fold) in Ubc9 transgenic mice compared with WT, but SUMO-2,3 HMW conjugates levels were only modestly (2-fold or less) increased in transgenic mice. After MCAO surgery, not only SUMO-1 conjugation levels, but also SUMO-2,3 conjugation levels in transgenic mice were significantly higher compared with WT (representative immunoblots are shown in Fig. 3B top panel, and density analyses, n = 10 for WT, n = 8 for N2, N = 8 for H3, n = 7 for K5 and n = 4 for G3, are shown in Fig. 3B bottom panel). The Western blot data were corroborated by immunohistochemical studies shown in Figure 3C. Condensed nuclear SUMO-1 (SUMO-2,3 to a lesser extent) expression considered as conjugated forms [14] was much higher in the brain (both ipsilaterally and contralaterally) of transgenic mouse (H3) compared to WT. Unlike WT mice, SUMO-1 as well as SUMO-2,3 and Ubc9 were expressed uniformly in the whole brain of transgenic mice (Figs. S3, S4, S5, S6, and S7). The major SUMO-1 and SUMO-2,3 immunoreactive cells in cerebral cortex were predominantly neurons (NeuN- and Parvalbumin-positive) (Fig. 3C).

### SUMO-1 and SUMO-2,3 conjugation levels have a positive linear correlation with Ubc9 levels and a negative linear correlation with brain infarction volumes in WT and Ubc9 transgenic mice

**Figure 4:**
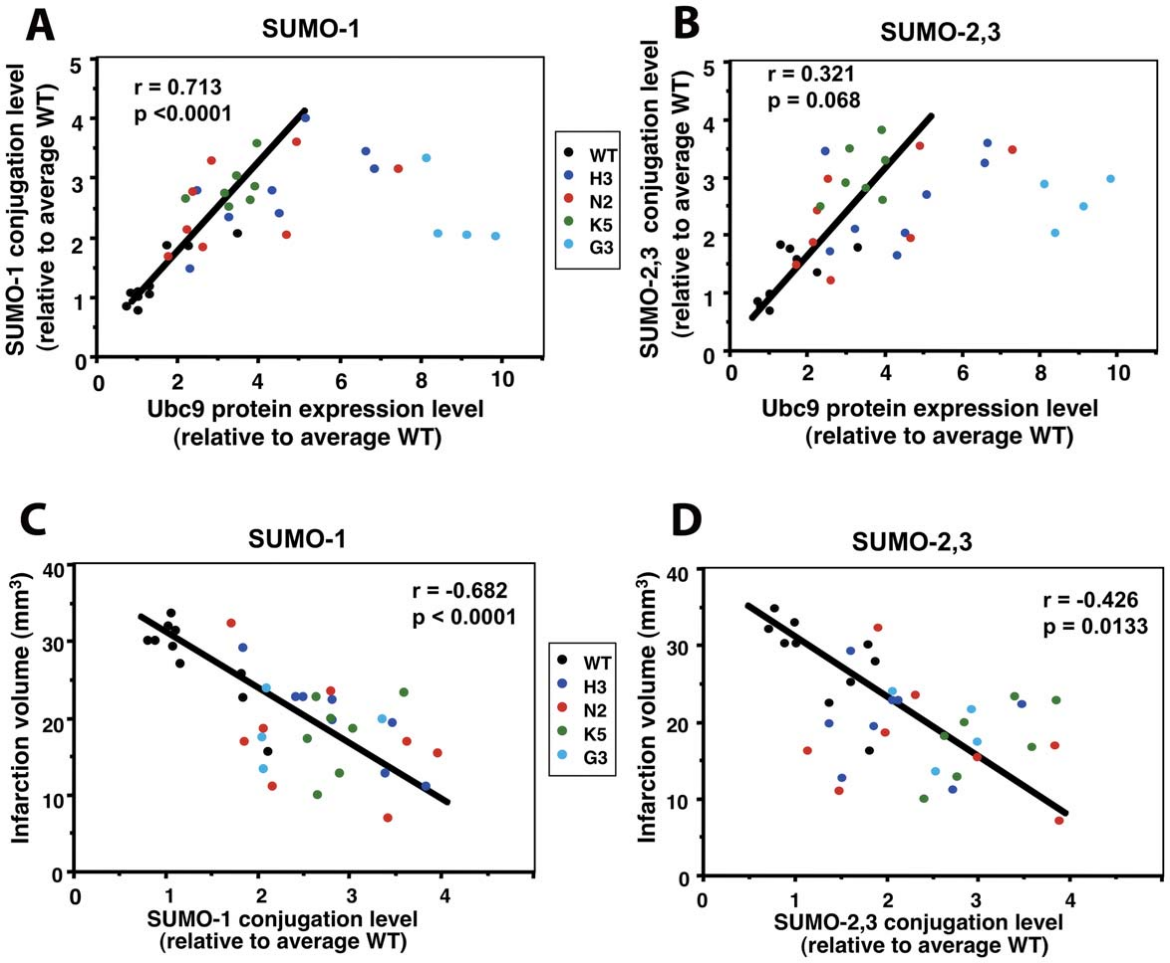
Correlation of SUMO-1 HMW-conjugates or SUMO-2,3 HMW- conjugates with either Ubc9 level or infarction volume. (A) The brain SUMO-1 HMW-conjugates level (y-axis) after 24 h pMCAO was plotted against the Ubc9 level (x-axis) in the same sample. Animals whose Ubc9 levels were under 5-fold the wild type show a significant correlation. (n = 42; r: Pearson correlation coefficient = 0.713, p<0.0001). (B) The brain SUMO-2,3 HMW-conjugates level (y-axis) was plotted against the Ubc9 level (x-axis) in the same sample. Animals whose Ubc9 levels were under 5-fold the wild type show only a trend toward correlation. (n = 42; r = 0.321, p = 0.068). (C) The brain infarction volumes (y-axis) after 24 h pMCAO were plotted against their SUMO-1 HMW-conjugates levels (x-axis). (n = 42; r = −0.682, p<0.0001). (D) The brain infarction volumes (y-axis) after 24 h pMCAO were plotted against their SUMO-2,3 HMW-conjugate levels. (n = 42; r = −0.426, p = 0.0133).

We plotted the Ubc9 protein expression levels against SUMO-1 HMW conjugate levels (Fig. 4A), and against SUMO-2,3 HMW conjugate levels (Fig. 4B) in the brains of WT and transgenic mice that had been subjected to 24 h pMCAO. SUMO-1 conjugation levels have a linear correlation with Ubc9 expression levels (r = 0.713, p<0.0001), as long as Ubc9 expression levels are not more than 5-fold increased over the WT level. An increase of more than 5-fold in Ubc9 level degraded the linear correlation with SUMO-1 conjugation levels by becoming somewhat inhibitory (Fig. 4A). SUMO-2,3 conjugation levels showed only a trend toward a linear correlation with Ubc9 expression level (r = 0.321, p = 0.068) (Fig. 4B). Again, Ubc9 expression above 5-fold of the WT level did not increase SUMO-2,3 conjugation further. When the SUMO-1 and SUMO-2,3 conjugation levels (x-axis) were plotted against infarction volumes (y-axis), however, there were significant inverse linear correlations without any linear discontinuities (r = −0.682, p<0.0001 for SUMO-1; r = −0.426, p = 0.0133 for SUMO-2,3) (Figs. 4C, D).

## Discussion

Ubc9 is the single E2-conjugating enzyme in the SUMO pathway. We previously showed that Ubc9 levels were well correlated with SUMO-1 and SUMO-2,3 conjugation levels in hibernating squirrels and the human neuroblastoma cell line SHSY5Y, and those with higher SUMO conjugation levels were more tolerant to ischemic insult [14], [16]. In this study, we established several lines of Ubc9 transgenic mice whose Ubc9 expression levels were elevated globally at various levels (Fig. 1). These Ubc9 transgenic mice turned out to be significantly more resistant to pMCAO, an animal stroke model, than corresponding wild type animals (Fig. 2B). Higher Ubc9 levels in the brain resulted in lower infarction volumes by pMCAO (Fig. 2C). However, the linear correlation was lost in high Ubc9 expressing animals. As we expected, increased Ubc9 levels contributed to the increase of SUMO-1 and SUMO-2,3 conjugation levels in the brain. But again, the linear correlation was lost in high Ubc9 expressing animals (Figs. 4A, B), suggesting that Ubc9 levels are critical for increasing global SUMOylation at low concentrations, but cease to be a limiting factor above 5–fold the endogenous baseline where such levels became suppressive.

Inhibitory effects of a high concentration of E2 enzyme in protein Ubiquitination and NEDD (NEural precursor cell-expressed Developmentally Downregulated-8) ylation have been shown in vitro [23], [24], [25]. Recently, an elegant study showed that a dramatic structural remodeling of the SUMO E1 enzyme (open form to closed form) takes place during its thioester bond formation with SUMO protein [26]. Wang et. al. also reported that at high concentrations SUMO E2 (Ubc9) interacts with the free E1 heterodimeric enzyme (SAE1/SAE2) and prevents this conformational change thereby inhibiting SUMO adenylation and the following SUMO thioester formation [27]. All of these inhibition studies had been done in *in vitro* systems. Our results suggest that inhibition of SUMOylation by high concentrations of Ubc9 happens *in vivo* as well.

Although Ubc9 is known to be the only E2 conjugase, there have been many reports identifying that enzyme as a multifunctional protein that independent of its role as a SUMOylation conjugating enzyme can act, for example, as a regulator of nuclear transport [28], a transcriptional co-factor [29], [30], [31] or a helper for virus production [32]. Interestingly, the interpretation that Ubc9 was acting independently of SUMOylation was mostly based on the effect being found not only for a wild-type Ubc9, but also for the dominant negative Ubc9 mutant (Ubc9-DN) that was devoid of E2 conjugase activity [28], [29], [30], [31], [32], [33], [34], [35]. Since Ubc9-DN may also bind to SUMO E1 and inhibit SUMOylation in the same way as overexpressed Ubc9-WT, the effects observed may be related to inhibition of SUMOylation and thus not be independent of the SUMOylation pathway.

Regarding the relationship between global SUMOylation and brain infarct volumes, the Ubc9 thresholds appear to have little direct bearing beyond the levels of global SUMOylation that they catalyze (Figs. 4C, D). Thus, increased global SUMOylation provides *in vivo* protection against ischemic brain damage in mice, although whether the conjugation pattern in transgenic lines is modified remains to be clarified. In hibernating squirrels, the SUMO-conjugation levels increased up to 30 times the control level during the torpor phase, even though Ubc9 levels only increased 2- to 3-fold [14]. Identifying ways to increase SUMO conjugation to levels approaching those reached by hibernating squirrels, which have such extraordinary resistance to blood flow levels characteristic of a stroke's ischemic core, is a worthy goal. We have focused on HMW SUMO conjugation levels in this study, however, additional data with conditional and tissue specific interference with Ubc9 or with deconjugating enzymes of SUMO conjugates would be useful in understanding the physiological impact of the SUMO system's neuroprotective effects. SUMOylation broadly affects the biological network and is involved in many cellular processes including gene expression, remodeling of chromatin structure, protein-protein interactions, changes in protein function, changes in protein intracellular locations, signal transduction, and maintenance of genome integrity [7], [36]. In general, global SUMOylation is involved in maintenance of homeostasis under stress [6].

We are investigating various approaches to identify small molecules that optimize global SUMO conjugation levels in order to find the limits of the cytoprotection that this plurifunctional mechanism [6] can deliver. Global SUMOylation is controlled by a process that is inherently accessible to small molecule regulation and, as demonstrated by heat shock in which global SUMOylation (for SUMO-2,3) can be induced within 5 minutes [37], this putative therapeutic approach could be applied acutely. Further study of the therapeutic potential of this post-translational modification of network dynamics is warranted.

## Materials and Methods

### Plasmid construction

The mouse Ubc9 coding sequence was inserted between the Kpn1 and Xho1 sites of pCCALL2-anton ΔlacZ ([38] and modified in Dr. KS Lee's lab (NCI/NIH)). The Ubc9 gene was located under control of the CMV early enhancer/chicken β-actin (CAG) promoter and followed by the rabbit β-globin poly adenylation sequence.

### Generation of Ubc9 transgenic mice

Ubc9/pCCALL2-anton ΔlacZ was digested with Sca1 and SfiI and the fragment containing CMV early enhancer/chicken β-actin (CAG) promoter, Ubc9 coding sequence and β-globin poly A sites was purified and injected into fertilized eggs. Founder mice (C57BL/6 background) were screened for the presence of the transgene by Southern analysis using BamHI for DNA digestion and the Ubc9 coding sequence as a ^32^P-labelled probe. These transgenic mice were generated by the Laboratory Animal Science Program in NCI-Frederick.

### Genotyping

Mice were marked by ear tags and genotyped by touchdown PCR on tail DNA prepared by DNeasy Blood & Tissue kit (Qiagen). Part of the CAG promoter sequence (5′-gcgccggcaggaaggaaatg-3′) was used as a forward primer and part of the Ubc9 coding sequence (5′-ggtgatagctggcctccagtcc-3′) was used as a reverse primer. Only the transgene (CAG-Ubc9) is amplified as a 650 bp DNA fragment.

### Animals and experimental protocols

The National Institute of Neurological Disorders and Stroke Animal Care and Use Committee approved all experiments under the protocol #1268-09 (project title: Use of Ubc9 transgenic mice for pre-clinical study of stroke). An equal number of male and female adult mice (18 to 24 weeks old, weight 20 to 30 g) were used in these experiments. Focal cerebral ischemia was induced in mice by cauterizing the distal portion of the middle cerebral artery. A 1.5∼2.0 cm skin incision is made between the left eyeball and ear. Following temporal muscle incision, a craniectomy is performed at the skull base (2 mm in diameter) to expose, cauterize and transect the left middle cerebral artery (MCA) and its anterior branch (if available) at the level above the olfactory tract. If the brain surface was visibly damaged or if the middle cerebral artery had bled owing to incomplete artery occlusion/coagulation, the animal was excluded from the study. The full MCAO procedure will generally cause an infarction that occupies about one-third of the ipsilateral hemisphere. At 24 hrs after surgery, each animal is anesthetized with isoflurane, decapitated and the brain is promptly removed and frozen for the assessment of brain damage by histological analysis.

### Pathology, physiology, and phenotyping

Six animals (3 male, 3 female) each from WT, low Ubc9 expressing lines and high Ubc9 expressing lines, all of which were 21 weeks old were subjected to pathology and phenotyping analyses. The analyses were done in the Diagnostic & Research Service Branch/Office of Director/NIH under the supervision of Dr. Mark Bryant.

### Labeling and imaging of Ubc9, Sumo-1 and Sumo-2, 3 expressions in tissue sections

*Mapping Ubc9 expression:* Ten µm thick mouse brain coronal sections were immunoreacted for 1 hour at room temperature (RT) using a 1 µg/ml mixture of the following primary antibodies: rabbit IgG anti-Ubc9 (Abcam), chicken IgG (IgY) anti-GFAP (Abcam) to identify astrocytes, mouse IgG1 anti-CNPase (Millipore) to identify oligodendrocytes, and guinea pig IgG anti-parvalbumin (Synaptic Systems) to identify interneurons. The sections were then washed in PBS/BSA and immunoreacted using a 1 µg/ml mixture of the following fluorochrome-conjugated secondary antibodies: goat anti-rabbit IgG-Alexa Fluor 700, goat anti-chicken IgG (IgY)-Alexa Fluor 594, goat anti-mouse IgG1-Alexa Fluor 350 (all from Invitrogen) and donkey anti-guinea pig IgG-IRDye 800CW (Li-Cor Biosciences). After rinsing excess secondary antibodies, the potentially unbound F(ab′)2 antigen binding sites on the goat anti-mouse IgG1-Alexa Fluor 350 secondary antibody were blocked using 30 minute incubation at RT in 5 µg/ml unlabeled mouse IgG1 antibody purified from non-immunized mouse serum (Bethyl Laboratories) and the sections then stained with 1 µg/ml Alexa Fluor 488-conjugated mouse IgG1 anti-NeuN (Millipore), a pan-neuronal marker. The slides with labeled tissue sections were then coverslipped using Immu-Mount medium (Thermo Fisher Scientific) and imaged using a multi-channel wide field fluorescence microscope (see below).

*Mapping Sumo-1 and Sumo-2, 3 expressions:* The immunostaining protocol for Sumo-1 and Sumo-2, 3 was essentially the same as detailed above for Ubc9 immunostaining with the following modification: rabbit IgG anti-Sumo-1 (in-house developed antibody) was used instead of rabbit IgG anti-Ubc9 and rat IgG2a anti-Sumo-2, 3 (Sigma-Aldrich) was included in the primary antibody mixture, which was subsequently visualized using goat anti-rat Alexa Fluor 546 (Invitrogen).

*Imaging of immunofluorescence reactions:* All sections were imaged using a Axiovert 200M fluorescence microscope (Carl Zeiss) equipped with a 20× Plan-Apochromat (Phase-2) objective (Carl Zeiss), a high resolution ORCA-ER cooled digital camera (Hamamatsu Photonics) sensitive to a wide-spectrum of emission wavelengths, including those approaching infrared, a 100W mercury arc lamp (Carl Zeiss), and excitation/dichroic/emission filter sets (Semrock) optimized to detect the following fluorophores: Alexa Fluor 350, Alexa Fluor 488, Alexa Fluor 546, Alexa Fluor 594, Alexa Fluor 700 and IRDye 800CW. Each labeling reaction was captured using filtered light through an appropriate fluorescence filter set and the images individually digitized at 12-bit resolution using the Volocity imaging program (Improvision). An appropriate color table was applied to each image to either match its emission spectrum or to set a distinguishing color balance. The pseudocolored images were then converted into TIFF files, exported to Adobe Photoshop and overlaid as individual layers to create multi-colored merged composites.

### Cresyl violet staining

Frozen brain coronal sections (20 µm) from operated (24 h after MCAO) wild type and Ubc9 transgenic mice were fixed with paraformaldehyde vapor overnight. The sections on slides were treated with ethanol (30 min), hydrated sequentially (100% ethanol, 95% ethanol, 70% ethanol, and H_2_O), and stained with 0.5% cresyl violet in H_2_O/acetic acid for 6 min. After washing with H_2_O, the sections were dehydrated sequentially (75% ethanol, 95% ethanol, 100% ethanol, Histoc-Clear) and mounted with cover slide for longer storage.

### Assessment of infarct volumes

Quantification of the infarct area was performed on the cresyl-violet-stained sections (20 µm thickness and every 0.34 mm interval). The infarct area of each section was measured using the NIH Image J program, and corrected for the effect of edema by the following equation [39]: Corrected infarct area = infarct area×area of contralateral hemisphere/area of ipsilateral hemisphere. Infarct volume (mm^3^) was calculated for each animal by integrating the corrected infarct area with the distance between sections (0.34 mm) [40].

### Real-time PCR

Total RNA was isolated from tail or brain tissues of WT and transgenic mice by Trizol and RNeazy RNA isolation kit (Qiagen). In a given experiment, the same amount of RNA was used from each sample. The real-time PCR was performed by the one-step method using the QuantiTect SYBR Green RT-PCR Master Mix together with the QuantiTect RT Mix (Qiagen), according to the manufacturer's protocol. The mRNA level of Ubc9 was normalized with β-tubulin (using the Tubb4 primer set, Qiagen) as an internal control. The primers used for Ubc9 were sense 5′-gatgactatccgtcctcaccacc and antisense 5′-ggtgatagctggcctccagtcc. The delta Ct (threshold cycle) value (ΔCt) in each sample was normalized by the formula ΔCt = Ct Ubc9−Ct tubulin. The fold change was calculated as ( = 2^−ΔΔCt^), where ΔΔCt was the difference between a given sample and a wild type sample ( = ΔCt sample−ΔCt wild type).

### Western blot analysis

Frozen brains, kidneys or tails were crushed on dry ice to make powder, added to 2% SDS, 60 mM Tris-HCl (pH 6.8), 50 mM EDTA, protease inhibitor cocktail (Roche), 1 mM PMSF (Sigma) and 20 mM N-ethylmaleimide (Sigma), and homogenized either by MagNA Lyser (Roche) for 2 cycles of 6500 rpm×50 sec, or a tissue homogenizer for 10 sec. The homogenates were sonicated for 10 sec, heated for 10 min at 95°C and centrifuged at 15,000*g* for 10 min at 4°C. After the protein concentration was measured, the supernatant was heated again at 95°C for 5 min with 5% β-mercaptoethanol and 2% glycerol, and subjected (20 µg/lane) to SDS-PAGE (4% to 20% in Tris-glycine). Western blot analyses were performed using the following antibodies: rabbit monoclonal anti-Ubc9 (abcam), rabbit polyclonal anti-SUMO-1 and anti-SUMO-2/3 antibodies (both antibodies developed in-house), and mouse monoclonal anti-β-actin antibodies (Sigma). Intensities of bands were analyzed by the densitometry program ImageJ (NIH). For SUMO conjugates band analysis, the higher molecular weight area (100 ∼300 kDa) in each lane was cropped and analyzed.

### Statistics

The independent two sample t-test was used to compare the means of two groups of interest. Correlation between two variables of interest was obtained by using Pearson's correlation coefficient. All statistical analyses were performed by means of SAS (SAS institute Inc., Cary, NC) and all reported p-values are two-tailed. The significance level was P<0.05.

## Supporting Information

Figure S1 **Distribution of Ubc9 expression and localization in a whole brain section.** A whole coronal section of Ubc9 transgenic mouse brain (line H3) was immunostained for Ubc9 (red) and the following neuronal and glial markers: NeuN (green), a pan-neuronal marker; parvalbumin (purple), a marker for interneurons; GFAP (yellow), a marker for astrocytes; CNPase (blue), a marker for oligodendrocytes. Upper panel shows a whole brain section with all five immunostainings together. The bottom panels are enlarged images of cerebral cortex region (dashed boxed area in the top panel) showing individual cell types with Ubc9 expression. Ubc9 is abundantly expressed in most neurons, with astrocytes and oligodendrocytes exhibiting low expression levels of this protein.
(TIF)

Figure S2 **SUMO-1 and SUMO-2,3 conjugation levels in the brain from WT and Ubc9 transgenic mice with and without MCAO.** Representative immunoblots of whole SUMO-1 and SUMO-2,3 protein patterns in the brain extracts from the WT and transgenic mice without MCAO surgery (A) or that had been subjected to 24 h pMCAO (B). The top panels show Ubc9 levels and the bottom panels show actin levels.
(TIF)

Figure S3 **Distribution of neuronal cells in a whole coronal section of WT and Ubc9 transgenic (H3) mice that had been subjected to 24 h pMCAO.** NeuN (green), a pan-neuronal marker; parvalbumin (purple), a marker for interneurons.
(TIF)

Figure S4 **Distribution and intensity of SUMO-1 (conjugated and free form) in a whole coronal section of WT and Ubc9 transgenic (H3) mice that had been subjected to 24 h pMCAO.**
(TIF)

Figure S5 **Distribution and intensity of SUMO-2,3 (conjugated and free form) in a whole coronal section of WT and Ubc9 transgenic (H3) mice that had been subjected to 24 h pMCAO.**
(TIF)

Figure S6 **Merged with NeuN (green), parvalbumin (purple), SUMO-1 (orange), and SUMO-2,3 (yellow) in a whole coronal section of WT and Ubc9 transgenic (H3) mice that had been subjected to 24 h pMCAO.**
(TIF)

Figure S7 **Distribution and intensity of Ubc9 in a whole coronal section adjacent to the above (Figures S3, S4, S5, S6) of WT and Ubc9 transgenic (H3) mice that had been subjected to 24 h pMCAO.**
(TIF)
