# Supplementary figures and images for: Elevated Global SUMOylation in Ubc9 Transgenic Mice Protects Their Brains against Focal Cerebral Ischemic Damage
Source: PLoS One. 2011 Oct 7;6(10):e25852. doi: 10.1371/journal.pone.0025852 (PMC3189225; doi:10.1371/journal.pone.0025852)

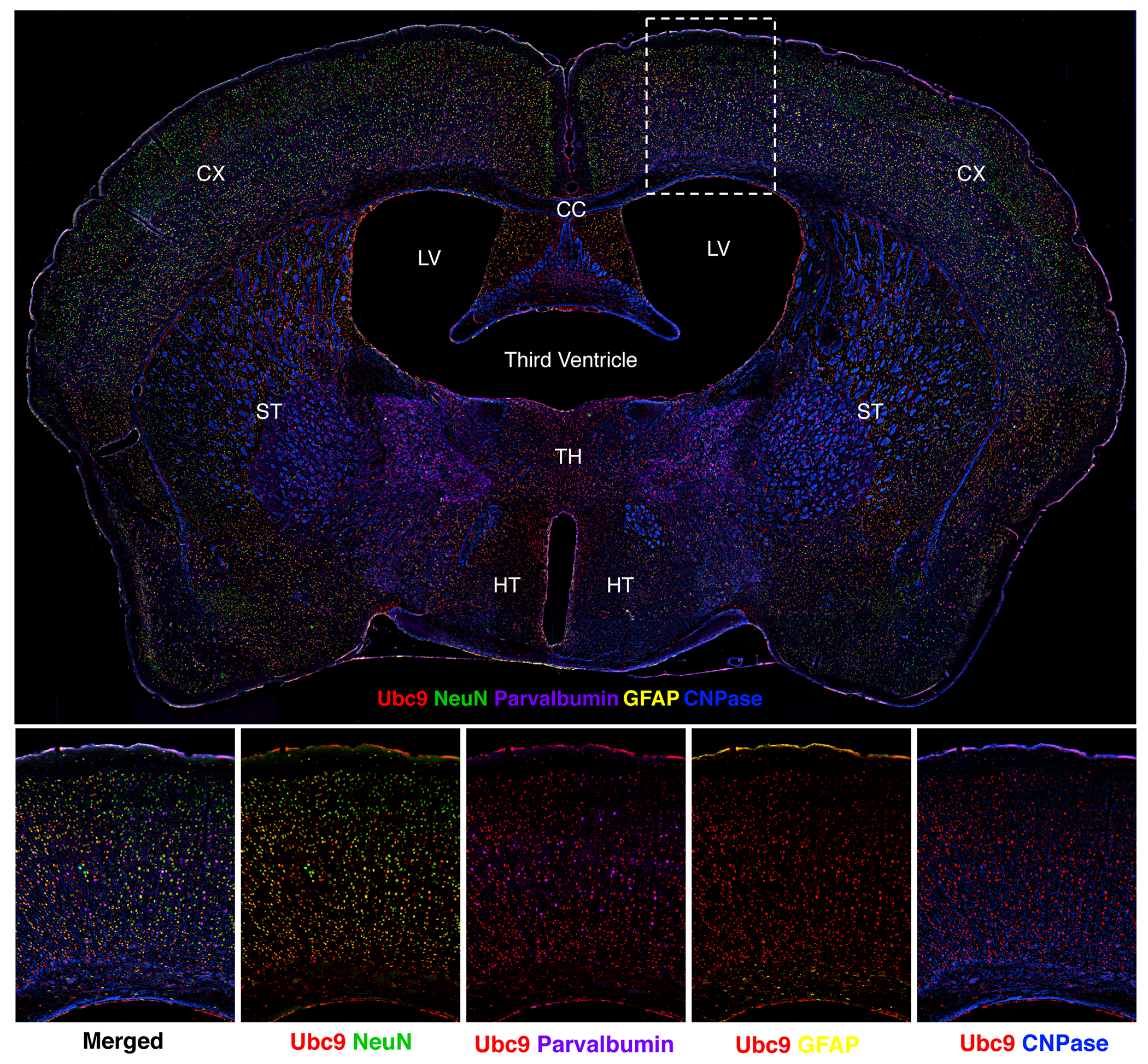

Supplement: Figure S1 — Distribution of Ubc9 expression and localization in a whole brain section. A whole coronal section of Ubc9 transgenic mouse brain (line H3) was immunostained for Ubc9 (red) and the following neuronal and glial markers: NeuN (green), a pan-neuronal marker; parvalbumin (purple), a marker for interneurons; GFAP (yellow), a marker for astrocytes; CNPase (blue), a marker for oligodendrocytes. Upper panel shows a whole brain section with all five immunostainings together. The bottom panels are enlarged images of cerebral cortex region (dashed boxed area in the top panel) showing individual cell types with Ubc9 expression. Ubc9 is abundantly expressed in most neurons, with astrocytes and oligodendrocytes exhibiting low expression levels of this protein. (TIF) [file pone.0025852.s001.tif]

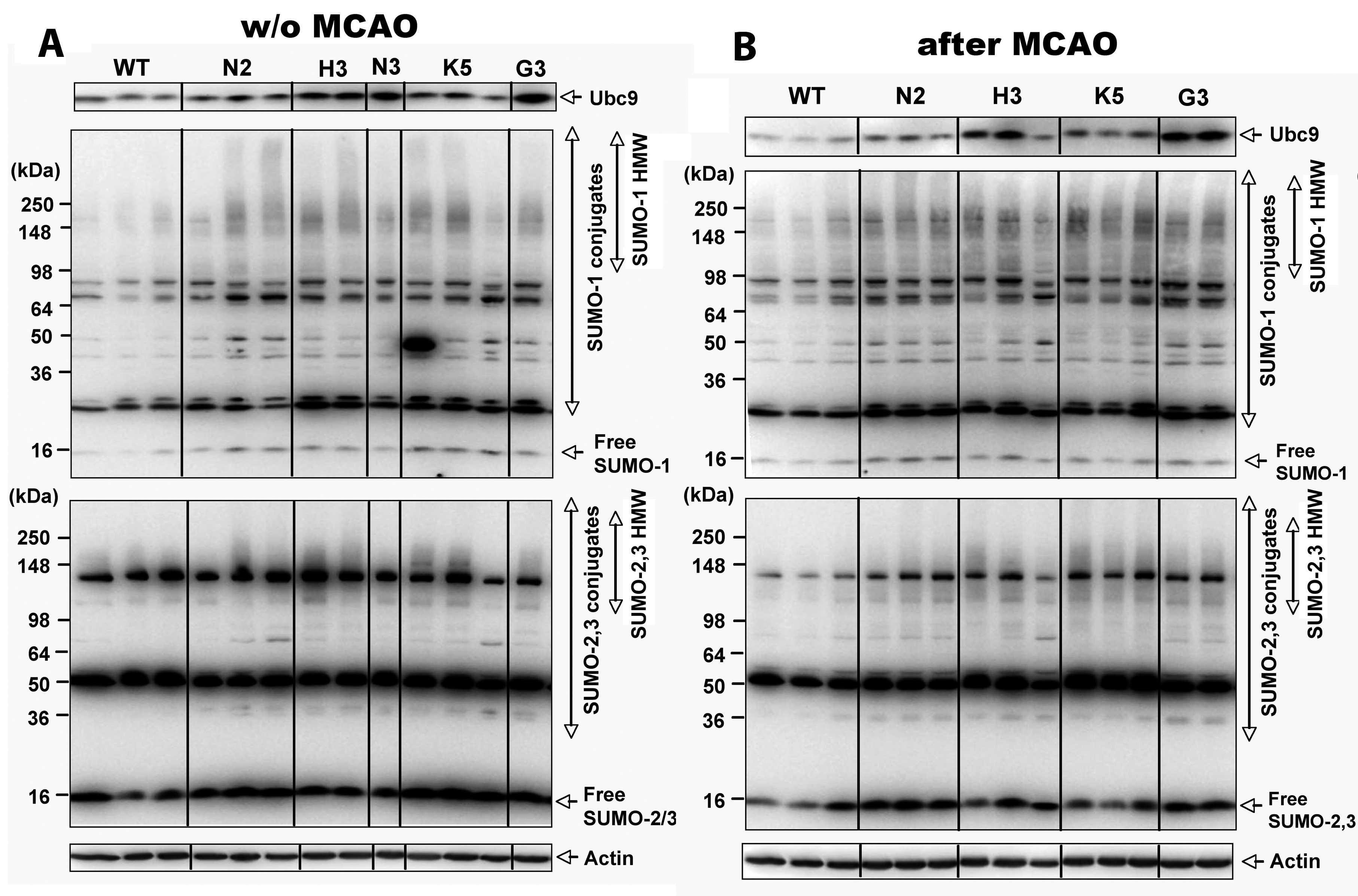

Supplement: Figure S2 — SUMO-1 and SUMO-2,3 conjugation levels in the brain from WT and Ubc9 transgenic mice with and without MCAO. Representative immunoblots of whole SUMO-1 and SUMO-2,3 protein patterns in the brain extracts from the WT and transgenic mice without MCAO surgery (A) or that had been subjected to 24 h pMCAO (B). The top panels show Ubc9 levels and the bottom panels show actin levels. (TIF) [file pone.0025852.s002.tif]

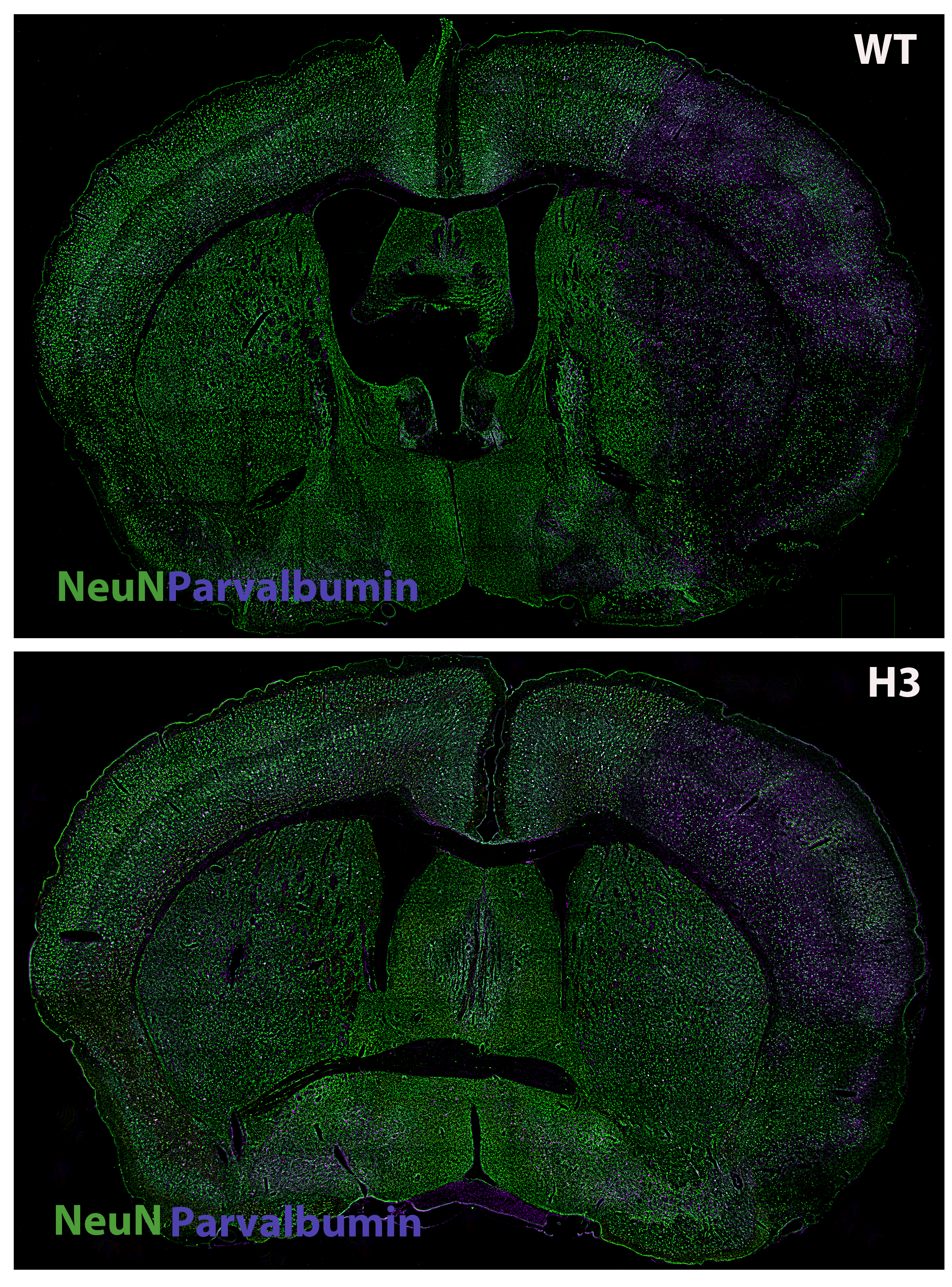

Supplement: Figure S3 — Distribution of neuronal cells in a whole coronal section of WT and Ubc9 transgenic (H3) mice that had been subjected to 24 h pMCAO. NeuN (green), a pan-neuronal marker; parvalbumin (purple), a marker for interneurons. (TIF) [file pone.0025852.s003.tif]

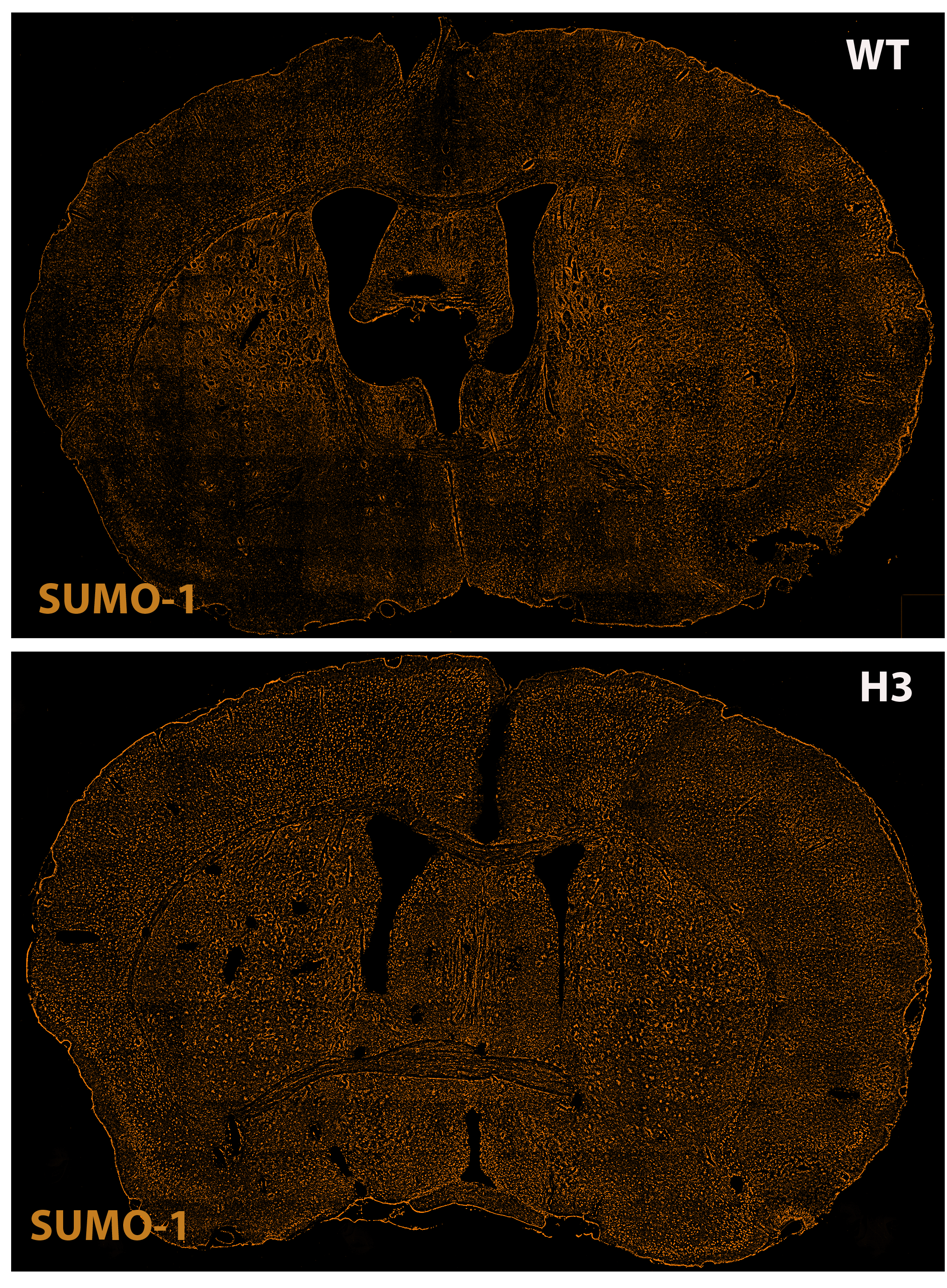

Supplement: Figure S4 — Distribution and intensity of SUMO-1 (conjugated and free form) in a whole coronal section of WT and Ubc9 transgenic (H3) mice that had been subjected to 24 h pMCAO. (TIF) [file pone.0025852.s004.tif]

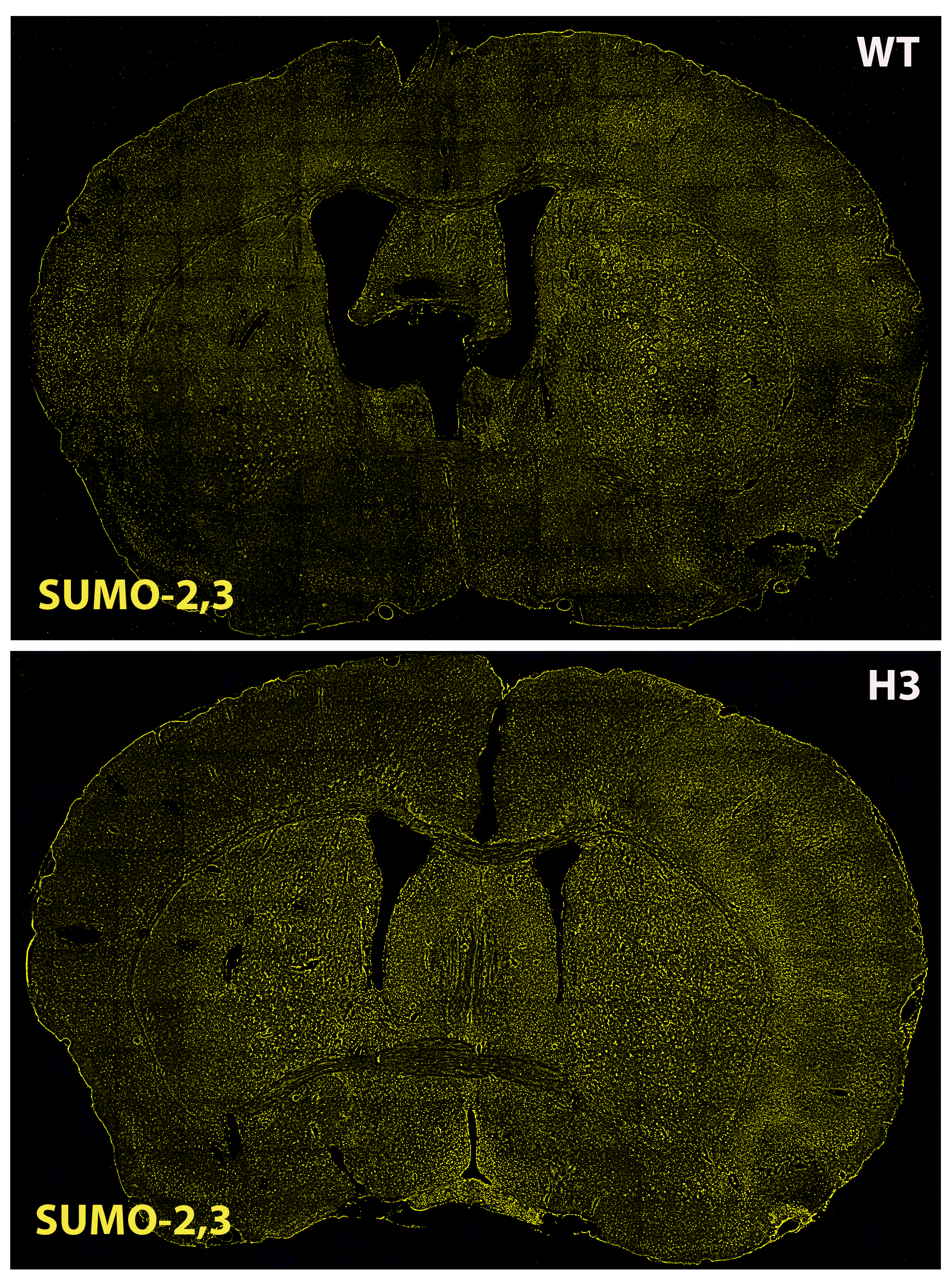

Supplement: Figure S5 — Distribution and intensity of SUMO-2,3 (conjugated and free form) in a whole coronal section of WT and Ubc9 transgenic (H3) mice that had been subjected to 24 h pMCAO. (TIF) [file pone.0025852.s005.tif]

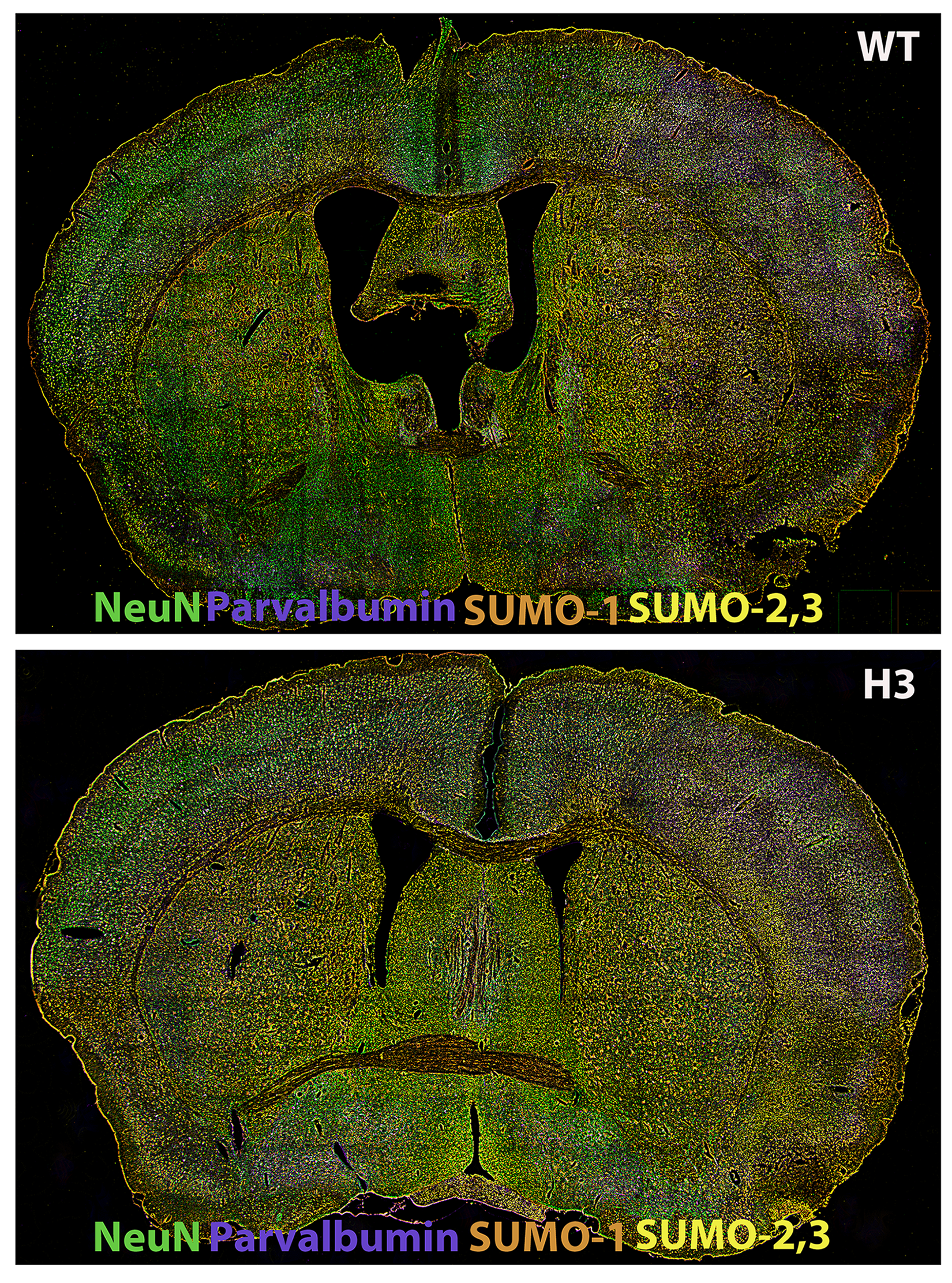

Supplement: Figure S6 — Merged with NeuN (green), parvalbumin (purple), SUMO-1 (orange), and SUMO-2,3 (yellow) in a whole coronal section of WT and Ubc9 transgenic (H3) mice that had been subjected to 24 h pMCAO. (TIF) [file pone.0025852.s006.tif]

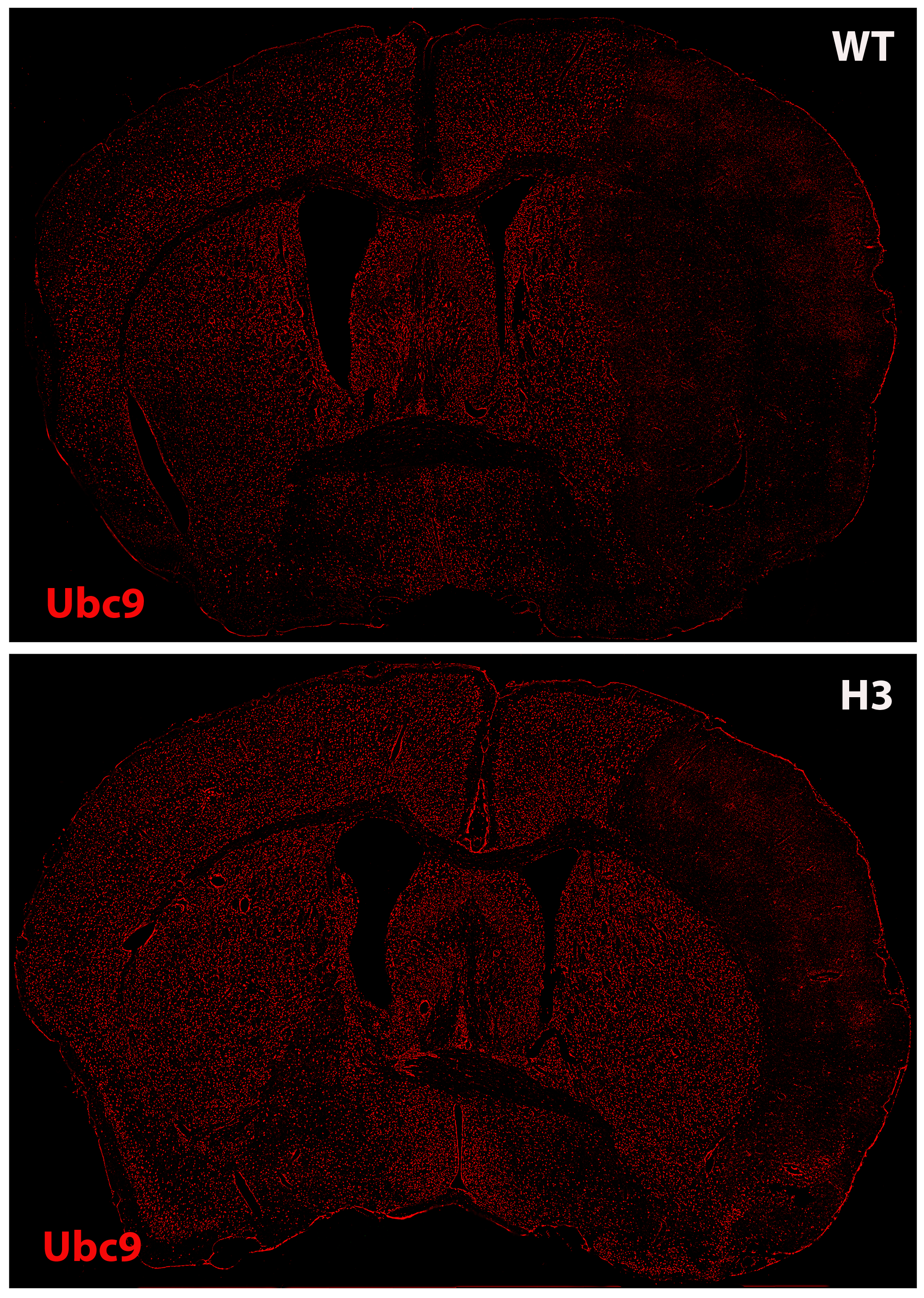

Supplement: Figure S7 — Distribution and intensity of Ubc9 in a whole coronal section adjacent to the above (Figures S3, S4, S5, S6) of WT and Ubc9 transgenic (H3) mice that had been subjected to 24 h pMCAO. (TIF) [file pone.0025852.s007.tif]
